# Quality of life and healthcare resource utilization among adult patients with short bowel syndrome: A mixed‐methods study leveraging an integrated database

**DOI:** 10.1002/ncp.70059

**Published:** 2025-11-11

**Authors:** Deborah Kuk, Brian Po‐Han Chen, Megan Gower, Michelle Kirby, Brian Terreri, Josh Feldman, Maggie McCue, Manpreet S. Mundi

**Affiliations:** ^1^ Inspire Arlington Virginia USA; ^2^ Takeda Pharmaceuticals U.S.A., Inc Lexington Massachusetts USA; ^3^ Division of Endocrinology, Diabetes, Metabolism, and Nutrition Mayo Clinic Minnesota Rochester Minnesota USA

**Keywords:** financial stress, healthcare resource utilization, parenteral nutrition, quality improvement, short bowel syndrome

## Abstract

**Background:**

Short bowel syndrome (SBS) is a chronic condition requiring parenteral nutrition (PN) support and multidisciplinary management. However, disparities in access to care and standardized treatment pathways, in addition to economic burden, remain considerable for patients. Understanding healthcare resource utilization (HCRU) and treatment patterns, especially in relation to patient‐reported outcomes (PROs), is critical for improving care.

**Methods:**

A cross‐sectional, multiphase study was conducted in 2023. This analysis focuses on phase 3, using a deidentified database that integrated claims and PROs from 68 patients in phase 2. Twenty‐three patients were included in the analytic cohort after applying additional criteria. Descriptive statistics summarized patient demographics, HCRU, and treatment patterns for the overall cohort and stratified by median SBS–quality of life (QoL) score.

**Results:**

The median age at SBS diagnosis was 35 years, and 91% of patients were female. Patients saw a median of 35 different healthcare providers and underwent approximately five procedures before diagnosis. In the 6 months after diagnosis, 39% had been hospitalized, and 26% had emergency room (ER) visits. Healthcare costs increased after diagnosis, with long‐term PN use accounting for 34% of total costs. Patients with worse QoL had a higher number of ER visits than patients with better QoL. Patients who self‐reported being treated by SBS specialists had lower healthcare costs than patients who did not receive specialized care.

**Conclusion:**

SBS is associated with high HCRU and costs, particularly for patients with poor QoL. Multidisciplinary care, especially from SBS specialists, may help reduce healthcare costs and improve patient outcomes.

## INTRODUCTION

Short bowel syndrome (SBS) is a rare, chronic, and debilitating condition most often caused by surgical resection of the small intestine in the treatment of inflammatory bowel disease, trauma, malignancy, radiation, or mesenteric ischemia.^1^ Patients with SBS face several complications resulting from altered bowel anatomy and physiology, the underlying disease, and its treatment, including the need for parenteral nutrition (PN) and the use of a central venous catheter.^2^ The complex individualized management of SBS often requires the expertise of several specialist healthcare professionals (HCPs), including registered dietitians, physicians, registered nurses, psychologists, and social workers, who work as a multidisciplinary team to achieve the best outcomes. However, disparities of care remain in terms of access to a multidisciplinary treatment approach.

Complications arising from SBS and PN frequently result in hospitalizations and emergency room (ER) visits, with acute issues including diarrhea, electrolyte imbalances, gallstones, kidney stones, and catheter‐related complications.^3^ Gastric hypersecretion, which may cause esophagitis and ulcers, is common, and diarrhea can be caused by multiple factors such as rapid transit, bacterial overgrowth, and fat malabsorption. Bacterial overgrowth contributes to bloating, discomfort, and further malabsorption, and patients with SBS are at increased risk of micronutrient deficiencies, particularly fat‐soluble vitamins, as well as metabolic bone diseases like osteoporosis and osteomalacia. Although the benefits of nutrition support in managing SBS have been well documented, the use of home parenteral nutrition (HPN) has declined since 1992.^4^ Studies indicate that patients dependent on PN often experience reduced quality of life (QoL) owing to complications such as infections, catheter‐related issues, and metabolic imbalances.^5^ PN is essential for nutrient delivery, but its long‐term use is associated with increased healthcare costs and hospitalizations, further impacting patients' overall well‐being.^6^, ^7^, ^8^, ^9^ Addressing these challenges is critical for improving outcomes.

The SBS patient journey is often characterized by a lack of psychosocial, medical, and financial support, especially because of the rarity of SBS, which further limits HCPs' experience with patients with SBS.^10^ Despite the availability of multiple published guidelines^11^, ^12^, ^13^ for diagnosing and managing SBS, treatment algorithms and care pathways are often not followed, contributing to increased disease burden. A substantial economic burden has also been reported by adult patients who need HPN. A study in 2021 reported that the overall mean cost for SBS hospitalizations was $34,130 (2014 costs; all costs are given in US dollars), with costs of care higher for patients with sepsis, liver disease, or severe malnutrition.^14^ Another analysis identified a disparity in healthcare coverage in rural vs urban areas, with few HCPs managing large patient groups over a long period of time, and approximately half of the patients having to travel between 10 and 100 miles for HPN‐related management.^15^

This study is part of a multiphase SBS research program combining a quantitative survey with medical and pharmacy claims data. Phases 1 and 2 used qualitative and quantitative surveys to explore the real‐world impact of SBS, focusing on its physical limitations, effects on QoL, social determinants of health (SDOH), and mental health for patients and caregivers. The present phase (phase 3) aims to quantify the healthcare resource utilization (HCRU) and treatment patterns in patients with SBS by integrating claims data with patient‐reported outcomes (PROs).

## METHODS

### Study design

The study was a cross‐sectional, multiphase, mixed‐methods investigation conducted between March 2023 and September 2023. It comprised three distinct phases: qualitative interviews (phase 1), quantitative surveys (phase 2),^16^ and analysis of a database containing claims (phase 3). The research presented here focuses on the findings from phase 3. Using proprietary privacy‐preserving tokenization methods, participants for phase 2 were linked through an open‐claims database. This tokenization process ensured patient privacy through deidentification while allowing surveys and claims to be linked securely, enabling more comprehensive insights to be gained.

### Ethical compliance

This study (Protocol # CCR‐2022‐200302) received an exemption from Western Copernicus Group Institutional Review Board under 45 CFR § 46.104(d)(2). All participants provided informed consent and Health Insurance Portability and Accountability Act (HIPAA) authorizations, ensuring confidentiality, minimal risk, and access to protected health information for research purposes.

### Study setting and patient population

**Figure 1:**
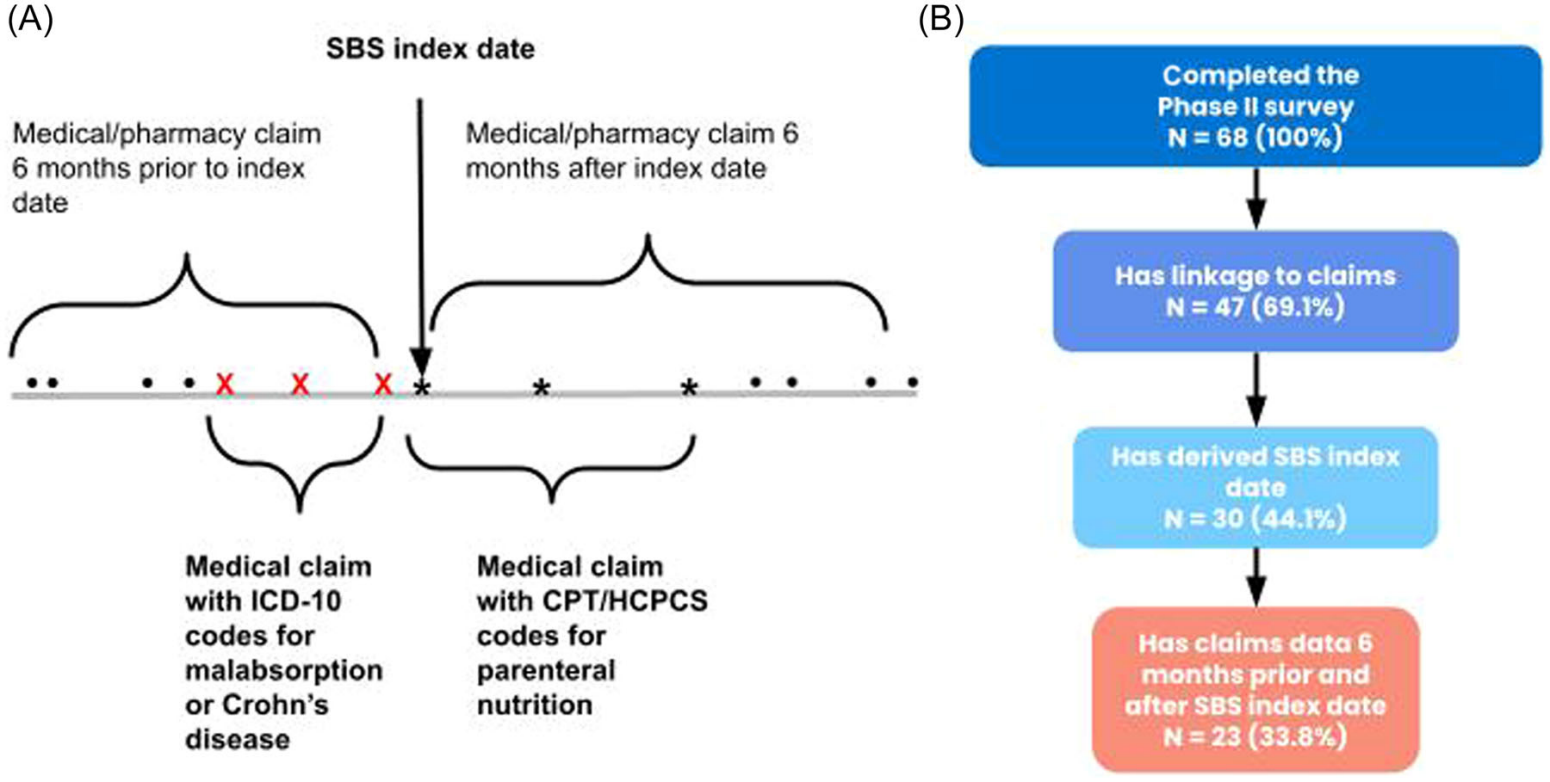
Analytic cohort criteria and cohort funnel in the study, including (A) an illustrative example of the analytic cohort criteria and definition of the index date and (B) the analytic cohort funnel for phase 3 of the study. In (A), black dots indicate medical or pharmacy claims that contain neither an *ICD‐10* code for malabsorption/Crohn's disease nor a claim with a *CPT*/HCPCS code for PN. *CPT*, *Current Procedural Terminology*; HCPCS, Healthcare Common Procedure Coding System; *ICD‐10*, *International Statistical Classification of Diseases and Related Health Problems, 10th Revision*; SBS, short bowel syndrome.

Patients were included if they were aged ≥18 years; resided in the United States; had participated in the previous survey phase (phase 2); had medical claims with *Current Procedural Terminology* (*CPT*)/Healthcare Common Procedure Coding System (HCPCS) code(s) for PN (Supporting Information S1: Table S1) that occurred during or after a claim with an *International Statistical Classification of Diseases and Related Health Problems, 10th Revision* (*ICD‐10*) code for malabsorption or Crohn's disease (Supporting Information S1: Table S2); and had claims data available for 6 months before and 6 months after their SBS diagnosis date. No *ICD‐10* code existed for SBS at the time of the analysis, so the index date of SBS diagnosis was the first date of service for parenteral infusion that occurred during or after a claim for malabsorption or Crohn's disease (Figure 1A).

### Brief description of phase 1 interviews and phase 2 survey

Phase 1 of the study included 1‐h qualitative interviews among six patients who self‐reported SBS and four caregivers using a semistructured discussion guide and conducted via web‐based teleconferencing. Insights from the qualitative interviews informed development of a 30‐min quantitative survey for phase 2. The phase 2 survey explored experiences of patients and caregivers with a focus on the causes and diagnosis of SBS; treatment experiences; care by an SBS specialist; the impact of PN; mental health; and various dimensions of SDOH, including housing, employment, and transportation needs. Additionally, patients' QoL was evaluated using the validated Short Bowel Syndrome–Quality of Life (SBS‐QoL) tool.^17^

### Data source

For a subgroup of 68 patients who participated in phase 2 of the study, survey results were linked with deidentified retrospective longitudinal medical and pharmacy claims records using an open‐claims database (Kythera Labs). The Kythera Labs open‐claims database^18^ contains data for >310 million patients and 9.7 billion healthcare claims, covering 79% of all US patients. The claims data cover 3 million practitioners, 400,000 organizations, and 1.2 million facilities.

### Data analysis

Descriptive statistics were generated to evaluate the primary and secondary objectives. For continuous variables, the median and interquartile range (IQR) are presented, whereas categorical variables were described using counts and percentages. Patient demographics such as age and geographic region were summarized, along with number of diagnostic tests, comorbidities, relevant medications, and HCPs seen. The index date of SBS diagnosis was defined as the first date of service with a *CPT*/HCPCS code(s) for PN that occurred during or after a claim with an *ICD‐10* code for malabsorption or Crohn's disease. Follow‐up time was calculated as the time between the SBS diagnosis date and the date of last claim available. *ICD‐10* codes from medical claims were used to calculate Charlson comorbidity index (CCI) scores. *ICD‐10* and *CPT*/HCPCS codes for hospitalizations and ER visits were assessed—for example, insertion of infusion device, bypass ileum to cutaneous, and change feeding device. Demographics, HCRU, and treatment patterns were stratified into better or worse QoL by dichotomizing the cohort using the median SBS‐QoL score obtained during phase 2. SBS‐QoL scores can range from 0 to 170, with a lower score indicating better QoL.

Owing to the small cohort size, only univariate analyses were conducted, with results reported for the entire patient cohort. The count and/or proportion of patients with missing data for each variable is reported. Patients had no missing survey data, and patients with any missing claims data were not excluded. Analysis for this study was conducted using R (version 4.2.1).

## RESULTS

Sixty‐eight patients with SBS completed the quantitative survey in phase 2 of this study. Forty‐seven of these patients (69%) were linked to medical and pharmacy claims. No significant differences in gender, age, highest education level, or ethnicity were found between those with linked claims (*n* = 47) and those without linked claims (*n* = 21). Thirty of the 47 patients had a derived SBS index date as defined in the Methods section. Twenty‐three patients in the analytic cohort had available claims data dating to 6 months before and 6 months after their derived SBS diagnosis date (Figure 1B).

In the analytic cohort, median age at SBS diagnosis was 35.0 years (IQR, 29.0–42.0), 91.3% (*n* = 21) of the cohort was female, and 34.8% (*n* = 8) of the cohort lived in the Midwest region of the United States. Median follow‐up time between SBS diagnosis and last claim was 73.0 months. Median follow‐up time from first claim to last claim date was 96.2 months (Table 1). There were 26,537 medical and pharmacy claims in total across the cohort, with a median of 1023.0 claims per patient. Claims associated with hospitalizations constituted 0.7% of all claims, and 22% of all claims were outpatient claims.

**Table 1:** Patient demographics and disease characteristics of patients with SBS.

Characteristic	*N* = 23
Current age, median (IQR), years	39.0 (34.0–45.0)
Current age group, *n* (%)	
18–35 years	7 (30.4)
36–45 years	11 (47.8)
46–55 years	2 (8.7)
56–65 years	2 (8.7)
66+ years	1 (4.3)
Age at SBS diagnosis, median (IQR), years	35.0 (28.0–44.0)
Age group at SBS diagnosis, *n* (%)	
18–35 years	13 (56.5)
36–45 years	5 (21.7)
46–55 years	3 (13.0)
56–65 years	1 (4.3)
66+ years	1 (4.3)
Gender as reported in survey, *n* (%)	
Female	21 (91.3)
Male	2 (8.7)
Region as reported in survey, *n* (%)	
Midwest	8 (34.8)
Northeast	6 (26.1)
South	4 (17.4)
West	5 (21.7)
Follow‐up time from SBS diagnosis, median (IQR), months	73.0 (28.6–81.9)

Abbreviations: IQR, interquartile range; SBS, short bowel syndrome.

### Overall HCRU

Patients saw a median of 35.0 different HCPs and underwent approximately five procedures before or during their SBS diagnosis (Table 2). Over half (56.5%) of the cohort had a mild CCI score (1–2), and 17.4% had none of the comorbidities that are given in the CCI. The median SBS‐QoL score was 123.4. Nine out of 23 patients had at least one hospitalization in the 6‐month period after the index date, with a median of one hospitalization per patient. Most, if not all, procedures associated with the hospitalizations were related to SBS, and most diagnosis codes were related to malnutrition, infection, dehydration, and procedural complications (Supporting Information S1: Tables S3, S4). Six patients had at least one ER visit in the 6‐month period after the index date, with a median of 2.5 visits per patient. Procedures and diagnosis codes recorded for ER visits were similar to those recorded for hospitalizations (Supporting Information S1: Tables S5, S6).

**Table 2:** Comorbidities and HCRU of patients with SBS.

Characteristic	*N* = 23
SBS‐related procedures undergone before or during SBS diagnosis, median (IQR)	5.0 (2.0–6.0)
Unknown	8
HCPs seen before or during SBS diagnosis, median (IQR)	35.0 (7.0–75.0)
Preexisting comorbidities, median (IQR)	1.0 (1.0–2.0)
CCI score, median (IQR)	1.0 (1.0–3.0)
CCI score, *n* (%)	
Mild (CCI 1–2)	13 (56.5)
Moderate (CCI 3–4)	3 (13.0)
Severe (CCI ≥ 5)	3 (13.0)
No comorbidities	4 (17.4)
SBS‐QoL score as reported in survey, median (IQR)	123.4 (111.3–145.3)
ER visits 6 months before or during SBS diagnosis, median (IQR)	2.5 (1.8–3.3)
Unknown	19
Hospitalizations 6 months before or during SBS diagnosis, median (IQR)	3.0 (3.0–4.0)
Unknown	18
ER visits up to 6 months after SBS diagnosis, median (IQR)	2.5 (1.3–4.5)
Unknown	17
Hospitalizations up to 6 months after SBS diagnosis, median (IQR)	1.0 (1.0, 3.0)
Unknown	14
All‐cause paid amount 6 months before SBS diagnosis,^a^ median (IQR), $	7635.00 (449.00–31,004.00)
Unknown	6
All‐cause paid amount 6 months after SBS diagnosis,^b^ median (IQR), $	72,849.00 (7435.00–199,015.00)
Unknown	3

Abbreviations: CCI, Charlson comorbidity index; ER, emergency room; HCP, healthcare provider; IQR, interquartile range; PN, parenteral nutrition; SBS‐QoL, Short Bowel Syndrome‐Quality of Life tool; SBS, short bowel syndrome.

^a^ Eight patients had cost information on PN. Median (IQR) all‐cause paid amount 6 months before SBS diagnosis in this subgroup was $22,766.00 ($401.00–$108,235.00).

^b^ Median (IQR) all‐cause paid amount 6 months after SBS diagnosis for the eight patients who had cost information on PN was $234,398.00 ($174,617.00–$302,420.00). Median (IQR) PN paid amount 6 months after SBS diagnosis was $80,089.00 ($36,369.00–$116,319.00), approximately 34% of the all‐cause paid amount.

### Dependence on PN

Patients in this study received PN well after their index SBS diagnosis date. In total, 6474 claims had a code for PN across the 23 patients, and the distribution of the 6474 submitted claims for PN after diagnosis was 26% for <1 year after SBS, 18% for 1–2 years, 37% for 3–5 years, and 19% for >5 years, respectively.

### Cost to payers

The median all‐cause paid amount (ie, the amount paid by patient insurance plans) 6 months before SBS diagnosis was $7635.00, and the median all‐cause paid amount 6 months after SBS diagnosis was $72,849.00. Only 8 of 23 patients had a paid amount recorded for PN 6 months after SBS diagnosis. In this subgroup of eight patients, the median all‐cause paid amount 6 months before SBS diagnosis was $22,766.00, and the all‐cause paid amount 6 months after SBS diagnosis was $234,398.00. The median paid amount of PN alone in these eight patients was $80,089.00. This indicates that, in this study cohort, PN accounts for about 34% of all‐cause paid amount and median all‐cause cost 6 months after SBS diagnosis. When patients were stratified according to whether they had been treated by an SBS specialist, those treated by an SBS specialist (*n* = 13) had lower all‐cause paid amounts in the 6‐month period before ($454.30 vs $9858.00) and after ($39,020.76 vs $109,106.70) SBS diagnosis than those not treated by an SBS specialist (*n* = 10).

### Stratified analysis by QoL

Patients were stratified by median SBS‐QoL score (≤123 and >123), with a lower score indicating better QoL. Eleven patients were in the better QoL group (SBS‐QoL ≤123), and 12 patients were in the worse‐QoL group (SBS‐QoL >123). Patients with better QoL were slightly younger at SBS diagnosis than patients with worse QoL (33 vs 36 years). Both groups had a similar number of claims per patient and a similar amount of follow‐up time between the first and last claim. Median follow‐up time from SBS diagnosis date was 6 months longer in the worse‐QoL group than in the better‐QoL group (74 vs 68 months) (Table 3).

**Table 3:** Patient demographics and disease characteristics of patients with SBS stratified by better vs worse QoL.

Characteristic	Better QoL (SBS‐QoL score ≤ 123), *n* = 11	Worse QoL (SBS‐QoL score > 123), *n* = 12
Current age, median (IQR), years	37.0 (31.0–45.0)	41.0 (36.5–47.0)
Current age group, *n* (%)		
18–35 years	5 (45.5)	2 (16.7)
36–45 years	4 (36.4)	7 (58.3)
46–55 years	1 (9.1)	1 (8.3)
56–65 years	1 (9.1)	1 (8.3)
66+ years	0 (0)	1 (8.3)
Age at SBS diagnosis, median (IQR), years	33.0 (25.0–44.0)	35.5 (30.0–43.0)
Age group at SBS diagnosis, *n* (%)		
18–35 years	7 (63.6)	6 (50.0)
36–45 years	2 (18.2)	3 (25.0)
46–55 years	1 (9.1)	2 (16.7)
56–65 years	1 (9.1)	0 (0)
66+ years	0 (0%)	1 (8.3)
Gender as reported in survey, *n* (%)		
Female	10 (90.9)	11 (91.7)
Male	1 (9.1)	1 (8.3)
Region as reported in survey, *n* (%)		
Midwest	5 (45.5)	3 (25.0)
Northeast	2 (18.2)	4 (33.3)
South	3 (27.3)	1 (8.3)
West	1 (9.1)	4 (33.3)
Claims per patient, median (IQR)	1010.0 (565.0–1635.0)	1031.5 (506.0–1219.5)
Claims per patient, *n* (%)		
1–249	1 (9.1)	1 (8.3)
250–499	0 (0)	2 (16.7)
500–999	4 (36.4)	2 (16.7)
1000+	6 (54.5)	7 (58.3)
Time between first and last claim, median (IQR), months	96.3 (90.3–98.1)	95.2 (87.0–102.7)
Follow‐up time from SBS diagnosis, median (IQR), months	68.4 (16.7–75.9)	73.6 (36.3–82.3)

Abbreviations: IQR, interquartile range; SBS, short bowel syndrome; SBS‐QoL, Short Bowel Syndrome–Quality of Life tool.

Patients with better QoL underwent fewer SBS‐related procedures before receiving their SBS diagnosis than those with worse QoL (2.0 vs 5.0). Both groups saw a similar number of HCPs before or during their SBS diagnosis and had the same median number of preexisting comorbidities (1.0 vs 1.0). Patients with worse QoL had a higher number of ER visits (3.0 vs 1.0) and hospitalizations (3.5 vs 1.0) in the 6‐month period before their SBS diagnosis than patients with better QoL. In the 6‐month period after receiving an SBS diagnosis, patients with worse QoL had slightly more ER visits than those with better QoL (3.0 vs 2.0). The all‐cause paid amount in the 6‐month period before receiving an SBS diagnosis was higher in the better‐QoL group than in the worse‐QoL group ($7635.00 vs $4614.65). The better‐QoL group also had a higher all‐cause paid amount in the 6‐month period after SBS diagnosis than the worse‐QoL group ($201,576.55 vs $26,982.88). PN paid amount was higher in the worse‐QoL group than in the better‐QoL group ($94,173.86 vs $58,032.06) (Table 4); however, the sample sizes for PN paid amount were limited.

**Table 4:** Comorbidities and healthcare resource utilization of patients with SBS stratified by better vs worse QoL.

Characteristic	Better QoL (SBS‐QoL score ≤ 123), *n* = 11	Worse QoL (SBS‐QoL score > 123), *n* = 12
SBS‐related procedures undergone before or during SBS diagnosis, median (IQR)	2.0 (1.0–6.0)	5.0 (4.0–10.0)
Unknown	2	6
HCPs seen before or during SBS diagnosis, median (IQR)	32.0 (7.0–60.0)	43.0 (9.0–105.5)
Preexisting comorbidities, median (IQR)	1.0 (1.0–2.0)	1.0 (1.0–3.0)
CCI score, median (IQR)	1.0 (1.0–2.0)	2.0 (1.0–3.5)
CCI score, *n* (%)		
Mild	7 (63.6)	6 (50.0)
Moderate	1 (9.1)	2 (16.7)
Severe	1 (9.1)	2 (16.7)
Unknown	2 (18.2)	2 (16.7)
ER visits 6 months before or during SBS diagnosis, median (IQR)	1.0 (1.0–1.0)	3.0 (2.0–4.0)
Unknown	10	9
Hospitalizations 6 months before or during SBS diagnosis, median (IQR)	1.0 (1.0–1.0)	3.5 (3.0–5.0)
Unknown	10	8
ER visits up to 6 months after SBS diagnosis, median (IQR)	2.0 (1.0–5.0)	3.0 (1.0–8.0)
Unknown	8	9
Hospitalizations up to 6 months after SBS diagnosis, median (IQR)	1.5 (1.0–3.5)	1.0 (1.0–3.0)
Unknown	7	7
All‐cause paid amount 6 months before SBS diagnosis, median (IQR), $	7635.00 (449.00–70,062.68)	4614.65 (409.30–20,431.00)
Unknown	2	4
All‐cause paid amount in the 6‐month period after SBS diagnosis, median (IQR), $	201,576.44 (8550.92–290,975.79)	26,982.88 (4089.20–109,106.70)
Unknown	1	2
PN paid amount in the 6‐month period after SBS diagnosis, median (IQR), $	58,032.06 (34,072.24–143,975.66)	94,173.86 (81,247.71–107,100.00)
Unknown	5	10

Abbreviations: CCI, Charlson comorbidity index; ER, emergency room; HCP, healthcare provider; IQR, interquartile range; PN, parenteral nutrition; SBS‐QoL, Short Bowel Syndrome–Quality of Life tool.

## DISCUSSION

This is a novel study that adopts a mixed‐method approach to quantify the HCRU and disease severity of patients with SBS using an integrated database in the United States. Other studies have evaluated pediatric patients with SBS^10^, ^19^, ^20^, ^21^ and patients with SBS outside the United States,^22^, ^23^ but our study quantifies HCRU and further stratifies adult patients with SBS by patient‐reported disease severity, which, to the best of our knowledge, other studies have not done. We found that patients in this study had high HCRU as evidenced in the number of HCPs seen during their SBS diagnostic journey, number of ER visits and hospitalizations, prolonged dependence on PN, and high cost to payers.

Our study findings align closely with existing literature and reinforce established associations between PN use and QoL in patients with complex conditions like SBS. Although directionality was not in the scope of our study, the results highlight that patients with worse QoL had more ER visits before and after their SBS diagnosis than those with better QoL. Hospitalizations in the 6‐month period before SBS diagnosis were higher in the worse QoL group than in the better QoL group. Hospitalizations were related to SBS, and most of the diagnosis codes recorded were malnutrition, infection, dehydration, and procedural complications (eg, complications of artificial openings of the digestive system). This corroborates findings from Siddiqui et al,^14^ in which 18.1% of patients hospitalized were reported to have dehydration, 38.3% received an anemia diagnosis owing to nutrition deficiencies, and 40.1% were diagnosed with some form of protein‐energy malnutrition. Overall, 10% of patients with SBS had a concurrent diagnosis of severe malnutrition.^14^

The consistency of our findings with studies from 2014 and 2023 supports our conclusions and further validates the broader understanding of the effect of PN on QoL, especially from the patient perspective.^24^, ^25^, ^26^

Winkler et al used an adaptation of Paterson's Shifting Perspectives Model of Chronic Illness and noted that patients often shift between viewing illness and wellness as the dominant lens impacting quality of life.^26^ When patients perceive their HPN dependence and its associated complications as central to their identity, one that is associated with significant burden, then they shift toward adopting an illness‐centered perspective. These individuals have lower quality of life and perceive that HPN is confining and associated with social isolation. They report lack of energy, physical weakness, and self‐pity. By contrast, when patients downplay the inconvenience of HPN and its complications, they tend to describe feeling strong, having good stamina and a positive attitude, and experiencing enjoyable social interactions, all of which contribute to higher perceived quality of life.

The present study observed that overall healthcare costs increased 10‐fold after diagnosis, with the cost of PN accounting for 34% of total healthcare costs. The median 6‐month paid amount for PN among the eight patients with available data was $80,089, which is likely to be an underestimate because most patients do not have cost information on PN. However, it is consistent with the annual HPN cost reported by Arhip et al ($103,000–$176,000 annually for US adults).^27^ PN use persisted for years after diagnosis in many cases, with more than half of all PN claims occurring at least 3 years after SBS diagnosis. The continued use of PN and complications of SBS leading to hospitalizations can result in a substantial financial burden for patients with SBS and their families.^14^, ^24^

Literature has suggested that patients with SBS benefit from care by a multidisciplinary team with expertise in intestinal failure that consists of a physician, a registered dietitian, a clinical pharmacist, nurses, supportive care members, and specialists.^25^ In addition, patients dependent on PN experienced improved outcomes with the establishment of nutrition support teams (NSTs). In a prospective 2‐year study of 375 patients receiving total PN, patients who were managed by an NST experienced a catheter complication rate of 3.7%, compared with a catheter complication rate of 33.5% for patients not managed by an NST.^28^ Another study calculated that $4.20 could be saved for every $1.00 assigned to NST implementation.^29^ Our study showed that the median 6‐month all‐cause paid amount after SBS diagnosis was $39,021 for the 13 patients treated by an SBS specialist, whereas those not treated by an SBS specialist (*n* = 10) had a median 6‐month all‐cause paid amount after SBS diagnosis of $109,107.

This study has several limitations that are commonly associated with survey and claims data analysis. One key limitation is the potential for recall bias that is inherent in self‐reported information, a frequent challenge in survey‐based research. The SBS‐QoL was collected cross‐sectionally, at different stages of each patient's SBS trajectory, which may have impacted our finding that the all‐cause paid amount in the 6‐month period before and after receiving an SBS diagnosis was higher in the better‐QoL group than in the worse‐QoL group. This finding may have also been confounded by receipt of care by an SBS specialist and PN use. The cutoff of 123 in the stratified analysis was based on the median because to our knowledge, there is no literature that suggests clinically meaningful cutoffs. Additionally, we acknowledge the small sample size of only 23 patients, who were predominately female and aged ≤45 years, and acknowledge that data may be missing or inaccurate, including coding errors in both medical and pharmacy claims. Virtual recruitment of the study required access to a mobile device or computer, which may have contributed to the increased number of younger participants and impacted generalizability of the study. The absence of an official diagnosis code for SBS in existing classifications (up to *ICD‐10*) at the time of the analysis poses another challenge, because patients may not have received a formal diagnosis of SBS and may instead identify with related conditions such as chronic nutrition malabsorption or HPN dependency. Although there was no SBS‐specific *ICD‐10* code at the time of the analysis, we are confident that the combination of self‐reported SBS and diagnosis and procedure codes related to SBS provided us with an analytic cohort of SBS patients.

However, this study offers distinct strengths. A primary advantage is that we linked PROs to medical and pharmacy claims data, providing a more comprehensive understanding of the patient's clinical experience. Open‐claims data allowed us to match patients across multiple payers with significantly larger population sizes and minimal time lag. This integration allowed patient‐reported disease severity and HCRU to be validated against actual claims, which strengthens the study findings. Given the limited patient population and the sparse data availability, matching patients to open‐claims data is even more important when studying a rare condition such as SBS. Moreover, the study used a mixed‐methods approach, with survey development guided by qualitative input from patients and expertise from a gastroenterologist. Virtual recruitment across the United States was facilitated by direct patient access from Inspire and the Oley Foundation partnership, expediting the recruitment process. Importantly, the study design enabled HCRU to be characterized by patient‐reported disease severity, further enhancing the insights into the healthcare needs of this underserved population.

## CONCLUSION

This novel study quantifies HCRU and disease severity for adult patients with SBS in the United States using a mixed‐methods approach. Findings reveal that patients with worse QoL experience higher HCRU, including increased hospitalizations and emergency visits, especially for conditions like malnutrition and dehydration. Postdiagnosis costs rose substantially, largely owing to prolonged PN dependence. Patients managed by SBS specialists showed reduced healthcare costs compared with those without specialized care, highlighting the value of multidisciplinary teams. Although limitations exist, integrating PROs with claims data in this study provides unique insights into the economic and clinical impact of SBS, highlighting the need for tailored care in this population.

## AUTHOR CONTRIBUTIONS

Deborah Kuk, Brian Po‐Han Chen, Megan Gower, Michelle Kirby, Brian Terreri, Maggie McCue, Josh Feldman, and Manpreet S. Mundi participated in the conceptualization and design of the study; Deborah Kuk analyzed the data and provided the methods; Deborah Kuk, Brian Po‐Han Chen, and Josh Feldman reviewed and verified the data; all authors interpreted the data; all authors critically revised the manuscript, read and approved the final manuscript, and agree to be fully accountable for ensuring the integrity and accuracy of the work.

## CONFLICT OF INTEREST STATEMENT

Deborah Kuk, Brian Po‐Han Chen, and Josh Feldman are Inspire employees. Megan Gower, Michelle Kirby, Brian Terreri, and Maggie McCue are employed by Takeda Pharmaceuticals U.S.A., Inc. and receive stock and/or stock options. Manpreet S. Mundi has received research grants from Nestlé and NorthSea. He is also on advisory boards for Baxter, Nutrishare, and Otsuka and part of an emerging experts' short bowel syndrome group with Zealand Pharma outside of the submitted work.

## Supporting information

Supplementary Tables
