# Supplementary material for: Quality of life and healthcare resource utilization among adult patients with short bowel syndrome: A mixed‐methods study leveraging an integrated database
Source: Nutr Clin Pract. 2025 Nov 11;41(4):1096–105. doi: 10.1002/ncp.70059 (PMC13419207; doi:10.1002/ncp.70059)
Supplement: Supplementary file 1 — Supplementary Tables [file NCP-41-1096-s001.docx]

# Supplementary tables

**TABLE S1** CPT/HCPCS Codes for Parenteral Nutrition used to define the analytic cohort and index SBS diagnosis.

| **CPT/HCPCS Code** | **Description** |
| --- | --- |
| B4168 | PARENTERAL NUTRITION SOLUTION; AMINO ACID, 3.5%, |
| B4197 | PARENTERAL NUTRITION SOLUTION; COMPOUNDED AMINO |
| B5000 | PARENTERAL NUTRITION SOLUTION COMPOUNDED AMINO A |
| E0779 | AMBULATORY INFUSION PUMP, MECHANICAL, REUSABLE, |
| B4220 | PARENTERAL NUTRITION SUPPLY KIT; PREMIX, PER DAY |
| B5100 | PARENTERAL NUTRITION SOLUTION COMPOUNDED AMINO A |
| S9367 | HOME INFUSION THERAPY, TOTAL PARENTERAL NUTRITION |
| S9377 | HOME INFUSION THERAPY, HYDRATION THERAPY; MORE T |
| B4180 | PARENTERAL NUTRITION SOLUTION; CARBOHYDRATES (DE |
| B4199 | PARENTERAL NUTRITION SOLUTION; COMPOUNDED AMINO |
| B9004 | PARENTERAL NUTRITION INFUSION PUMP, PORTABLE |
| B4185 | PARENTERAL NUTRITION SOLUTION, NOT OTHERWISE SPE |
| B4189 | PARENTERAL NUTRITION SOLUTION; COMPOUNDED AMINO |
| B4222 | PARENTERAL NUTRITION SUPPLY KIT; HOME MIX, PER D |
| B4224 | PARENTERAL NUTRITION ADMINISTRATION KIT, PER DAY |
| B5200 | PARENTERAL NUTRITION SOLUTION COMPOUNDED AMINO A |
| B9999 | NOC FOR PARENTERAL SUPPLIES |
| B4216 | PARENTERAL NUTRITION; ADDITIVES (VITAMINS, TRACE |
| C8957 | INTRAVENOUS INFUSION FOR THERAPY/DIAGNOSIS; INIT |
| B4193 | PARENTERAL NUTRITION SOLUTION; COMPOUNDED AMINO |
| B9006 | PARENTERAL NUTRITION INFUSION PUMP, STATIONARY |
| S9366 | HOME INFUSION THERAPY, TOTAL PARENTERAL NUTRITION |
| B4172 | PARENTERAL NUTRITION SOLUTION; AMINO ACID, 5.5% |
| B4178 | PARENTERAL NUTRITION SOLUTION: AMINO ACID, GREAT |
| E0791 | PARENTERAL INFUSION PUMP, STATIONARY, SINGLE OR |
| S9368 | HOME INFUSION THERAPY, TOTAL PARENTERAL NUTRITION |
| S9375 | HOME INFUSION THERAPY, HYDRATION THERAPY; MORE T |
| S9373 | HOME INFUSION THERAPY, HYDRATION THERAPY; ADMINI |
| S9374 | HOME INFUSION THERAPY, HYDRATION THERAPY; ONE LI |
| S9376 | HOME INFUSION THERAPY, HYDRATION THERAPY; MORE T |
| B4164 | PARENTERAL NUTRITION SOLUTION: CARBOHYDRATES (DE |
| B4176 | PARENTERAL NUTRITION SOLUTION; AMINO ACID, 7% TH |
| S9364 | HOME INFUSION THERAPY, TOTAL PARENTERAL NUTRITION |
| S9365 | HOME INFUSION THERAPY, TOTAL PARENTERAL NUTRITION |
| 99601 | HOME INFUSION/SPECIALTY DRUG ADMINISTRATION, PER VISIT (UP TO 2 HOURS) |
| 99602 | NUMBER OF ADDITIONAL HOURS FOR HOME INFUSION |
| 3E0336Z | INTRODUCTION OF NUTRITIONAL INTO PERIPH VEIN, PERC APPROACH |
| 3E0436Z | INTRODUCTION OF NUTRITIONAL SUBSTANCE INTO CENTRAL VEIN, PERCUTANEOUS APPROACH |
| 96360 | HYDRATION INFUSION |
| 96361 | HYDRATION INFUSION |

Abbreviations: Current Procedural Terminology; HCPCS, Healthcare Common Procedure Coding System SBS, short bowel syndrome.

**TABLE S2** ICD-10 Codes for malabsorption or Crohn’s disease used to define the analytic cohort and index SBS diagnosis.

| **ICD Code** | **Description** |
| --- | --- |
| K90.9 | intestinal malabsorption |
| K90.89 | other intestinal malabsorption |
| K91.2 | postsurgical malabsorption |
| K90.4 | Other malabsorption due to intolerance |
| K90.49 | Malabsorption due to intolerance, not elsewhere classified |
| K90.8 | Other intestinal malabsorption |
| K50.019 | Crohn’s disease of small intestine with unspecified complications |
| K50.80 | Crohn’s disease of both small and large intestine without complications |
| K50.819 | Crohn’s disease of both small and large intestine with unspecified complications |
| K50.90 | Crohn’s disease, unspecified, without complications |
| K50.919 | Crohn’s disease, unspecified, with unspecified complications |

Abbreviations: ICD-10, International Statistical Classification of Diseases and Related Health Problems 10th Revision; SBS, short bowel syndrome.

**TABLE S3**  Procedure codes recorded for hospitalizations among patients with hospitalizations in the 6-month period after the index date of SBS diagnosis.

| **Procedure code** | **Description** | **No. of patients** | **No. of claims** |
| --- | --- | --- | --- |
| 02HV33Z | INSERTION OF INFUSION DEV INTO SUP VENA CAVA, PERC APPROACH | 4 | 8 |
| 0D1B0Z4 | BYPASS ILEUM TO CUTANEOUS, OPEN APPROACH | 2 | 2 |
| 0DHA0UZ | INSERTION OF FEEDING DEVICE INTO JEJUNUM, OPEN APPROACH | 2 | 2 |
| 0D20XUZ | CHANGE FEEDING DEVICE IN UP INTEST TRACT, EXTERN APPROACH | 2 | 5 |
| 3E0H76Z | INTRODUCTION OF NUTRITIONAL INTO LOW GI, VIA OPENING | 2 | 2 |
| 3E0436Z | INTRODUCTION OF NUTRITIONAL INTO CENTRAL VEIN, PERC APPROACH | 2 | 5 |
| 0DPDXUZ | REMOVE OF FEEDING DEV FROM LOW INTEST TRACT, EXTERN APPROACH | 1 | 1 |
| 0DQ70ZZ | REPAIR STOMACH, PYLORUS, OPEN APPROACH | 1 | 1 |
| 0DBB0ZZ | EXCISION OF ILEUM, OPEN APPROACH | 1 | 1 |
| B518ZZA | FLUOROSCOPY OF SUPERIOR VENA CAVA, GUIDANCE | 1 | 1 |
| 0DTP0ZZ | RESECTION OF RECTUM, OPEN APPROACH | 1 | 1 |
| 0FT40ZZ | RESECTION OF GALLBLADDER, OPEN APPROACH | 1 | 1 |
| 3E0T3BZ | INTRODUCE LOCAL ANESTH IN PERIPH NRV, PLEXI, PERC | 1 | 1 |
| 3E0G76Z | INTRODUCTION OF NUTRITIONAL INTO UP GI, VIA OPENING | 1 | 2 |
| 3E03317 | INTRODUCE OTH THROMBOLYTIC IN PERIPH VEIN, PERC | 1 | 1 |
| BD16ZZZ | FLUOROSCOPY OF UPPER GI AND SMALL BOWEL | 1 | 1 |
| 0DTS0ZZ | RESECTION OF GREATER OMENTUM, OPEN APPROACH | 1 | 1 |
| 0JBD0ZX | EXCISION OF R UP ARM SUBCU/FASCIA, OPEN APPROACH, DIAGN | 1 | 4 |
| BD13ZZZ | FLUOROSCOPY OF SMALL BOWEL | 1 | 1 |
| B546ZZA | ULTRASONOGRAPHY OF RIGHT SUBCLAVIAN VEIN, GUIDANCE | 1 | 1 |
| 0JH63XZ | INSERTION OF VAD INTO CHEST SUBCU/FASCIA, PERC APPROACH | 1 | 3 |
| 0D9600Z | DRAINAGE OF STOMACH WITH DRAINAGE DEVICE, OPEN APPROACH | 1 | 1 |
| B548ZZA | ULTRASONOGRAPHY OF SUPERIOR VENA CAVA, GUIDANCE | 1 | 1 |
| 3E033XZ | INTRODUCTION OF VASOPRESSOR INTO PERIPH VEIN, PERC APPROACH | 1 | 1 |
| 05H533Z | INSERTION OF INFUSION DEV INTO R SUBCLAV VEIN, PERC APPROACH | 1 | 1 |
| 0DTL0ZZ | RESECTION OF TRANSVERSE COLON, OPEN APPROACH | 1 | 1 |
| 02H633Z | INSERTION OF INFUSION DEVICE INTO R ATRIUM, PERC APPROACH | 1 | 2 |
| 3E0G36Z | INTRODUCTION OF NUTRITIONAL INTO UP GI, PERC APPROACH | 1 | 2 |
| 0W9F3ZX | DRAINAGE OF ABDOMINAL WALL, PERCUTANEOUS APPROACH, DIAGN | 1 | 1 |
| B5181ZA | FLUOROSCOPY OF SUP VENA CAVA USING L OSM CONTRAST, GUIDANCE | 1 | 1 |
| 02PY33Z | REMOVAL OF INFUSION DEVICE FROM GREAT VESSEL, PERC APPROACH | 1 | 1 |
| 0JPT3XZ | REMOVAL OF VAD FROM TRUNK SUBCU/FASCIA, PERC APPROACH | 1 | 1 |
| 0DP6X0Z | REMOVAL OF DRAINAGE DEVICE FROM STOMACH, EXTERNAL APPROACH | 1 | 1 |
| 0DHA3UZ | INSERTION OF FEEDING DEVICE INTO JEJUNUM, PERC APPROACH | 1 | 1 |
| 0DNE0ZZ | RELEASE LARGE INTESTINE, OPEN APPROACH | 1 | 1 |
| 02PYX3Z | REMOVAL OF INFUSION DEV FROM GREAT VESSEL, EXTERN APPROACH | 1 | 3 |

Abbreviations: SBS, short bowel syndrome.

**Table S4** Primary ICD-10 codes recorded during hospitalizations among patients with hospitalizations in the 6-month period after the index date of SBS diagnosis.

| **Diagnosis code** | **Description** | **No. of patients** | **No. of claims** |
| --- | --- | --- | --- |
| A41 | Other sepsis | 2 | 3 |
| D68 | Other coagulation defects | 2 | 7 |
| E87 | Other disorders of fluid, electrolyte and acid-base balance | 2 | 2 |
| E86 | Volume depletion | 2 | 2 |
| T80 | Complications following infusion, transfusion and therapeutic injection | 2 | 4 |
| E43 | Unspecified severe protein-calorie malnutrition | 2 | 2 |
| F32 | Depressive episode | 2 | 3 |
| K94 | Complications of artificial openings of the digestive system | 2 | 6 |
| Q79 | Congenital malformations of musculoskeletal system, not elsewhere classified | 2 | 5 |
| Q43 | Other congenital malformations of intestine | 1 | 4 |
| G89 | Pain, not elsewhere classified | 1 | 1 |
| E03 | Other hypothyroidism | 1 | 1 |
| K59 | Other functional intestinal disorders | 1 | 1 |
| K50 | Crohn's disease [regional enteritis] | 1 | 1 |
| R10 | Abdominal and pelvic pain | 1 | 1 |
| T82 | Complications of cardiac and vascular prosthetic devices, implants and grafts | 1 | 1 |
| R64 | Cachexia | 1 | 1 |
| K43 | Ventral hernia | 1 | 2 |
| E27 | Other disorders of adrenal gland | 1 | 1 |
| J98 | Other respiratory disorders | 1 | 1 |
| T81 | Complications of procedures, not elsewhere classified | 1 | 2 |
| N17 | Acute kidney failure | 1 | 1 |
| F50 | Eating disorders | 1 | 3 |
| R50 | Fever of other and unknown origin | 1 | 2 |
| E44 | Protein-calorie malnutrition of moderate and mild degree | 1 | 3 |
| R00 | Abnormalities of heart beat | 1 | 2 |
| B37 | Candidiasis | 1 | 1 |
| C18 | Malignant neoplasm of colon | 1 | 1 |
| I82 | Other venous embolism and thrombosis | 1 | 2 |
| E66 | Overweight and obesity | 1 | 1 |
| K31 | Other diseases of stomach and duodenum | 1 | 1 |

Abbreviations: ICD-10, International Statistical Classification of Diseases and Related Health Problems 10th Revision; SBS, short bowel syndrome.

**TABLE S5** Procedure codes recorded for ER visits among patients with ER visits in the 6-month period after the index date of SBS diagnosis.

| **Procedure code** | **Description** | **No. of patients** | **No. of claims** |
| --- | --- | --- | --- |
| 02HV33Z | INSERTION OF INFUSION DEV INTO SUP VENA CAVA, PERC APPROACH | 3 | 7 |
| 3E0H76Z | INTRODUCTION OF NUTRITIONAL INTO LOW GI, VIA OPENING | 2 | 2 |
| 0D20XUZ | CHANGE FEEDING DEVICE IN UP INTEST TRACT, EXTERN APPROACH | 2 | 5 |
| 0DPDXUZ | REMOVE OF FEEDING DEV FROM LOW INTEST TRACT, EXTERN APPROACH | 1 | 1 |
| B518ZZA | FLUOROSCOPY OF SUPERIOR VENA CAVA, GUIDANCE | 1 | 1 |
| 3E0G76Z | INTRODUCTION OF NUTRITIONAL INTO UP GI, VIA OPENING | 1 | 2 |
| 3E03317 | INTRODUCE OTH THROMBOLYTIC IN PERIPH VEIN, PERC | 1 | 1 |
| BD16ZZZ | FLUOROSCOPY OF UPPER GI AND SMALL BOWEL | 1 | 1 |
| 0JBD0ZX | EXCISION OF R UP ARM SUBCU/FASCIA, OPEN APPROACH, DIAGN | 1 | 4 |
| BD13ZZZ | FLUOROSCOPY OF SMALL BOWEL | 1 | 1 |
| B546ZZA | ULTRASONOGRAPHY OF RIGHT SUBCLAVIAN VEIN, GUIDANCE | 1 | 1 |
| 0JH63XZ | INSERTION OF VAD INTO CHEST SUBCU/FASCIA, PERC APPROACH | 1 | 3 |
| 0D9600Z | DRAINAGE OF STOMACH WITH DRAINAGE DEVICE, OPEN APPROACH | 1 | 1 |
| 3E033XZ | INTRODUCTION OF VASOPRESSOR INTO PERIPH VEIN, PERC APPROACH | 1 | 1 |
| 0DHA0UZ | INSERTION OF FEEDING DEVICE INTO JEJUNUM, OPEN APPROACH | 1 | 1 |
| 05H533Z | INSERTION OF INFUSION DEV INTO R SUBCLAV VEIN, PERC APPROACH | 1 | 1 |
| 02H633Z | INSERTION OF INFUSION DEVICE INTO R ATRIUM, PERC APPROACH | 1 | 2 |
| 3E0436Z | INTRODUCTION OF NUTRITIONAL INTO CENTRAL VEIN, PERC APPROACH | 1 | 4 |
| 3E0G36Z | INTRODUCTION OF NUTRITIONAL INTO UP GI, PERC APPROACH | 1 | 2 |
| 0W9F3ZX | DRAINAGE OF ABDOMINAL WALL, PERCUTANEOUS APPROACH, DIAGN | 1 | 1 |
| B5181ZA | FLUOROSCOPY OF SUP VENA CAVA USING L OSM CONTRAST, GUIDANCE | 1 | 1 |
| 02PY33Z | REMOVAL OF INFUSION DEVICE FROM GREAT VESSEL, PERC APPROACH | 1 | 1 |
| 0JPT3XZ | REMOVAL OF VAD FROM TRUNK SUBCU/FASCIA, PERC APPROACH | 1 | 1 |
| 0DP6X0Z | REMOVAL OF DRAINAGE DEVICE FROM STOMACH, EXTERNAL APPROACH | 1 | 1 |
| 0DHA3UZ | INSERTION OF FEEDING DEVICE INTO JEJUNUM, PERC APPROACH | 1 | 1 |
| 02PYX3Z | REMOVAL OF INFUSION DEV FROM GREAT VESSEL, EXTERN APPROACH | 1 | 3 |

Abbreviations: ER, emergency room; SBS, short bowel syndrome.

**TABLE S6** Primary ICD-10 codes recorded during ER visits among patients with ER visits in the 6-month period after the index date of SBS diagnosis.

| **Diagnosis code** | **Description** | **No. of patients** | **No. of claims** |
| --- | --- | --- | --- |
| A41 | Other sepsis | 2 | 3 |
| D68 | Other coagulation defects | 2 | 7 |
| E87 | Other disorders of fluid, electrolyte and acid-base balance | 2 | 2 |
| E86 | Volume depletion | 2 | 2 |
| T80 | Complications following infusion, transfusion and therapeutic injection | 2 | 4 |
| K94 | Complications of artificial openings of the digestive system | 2 | 6 |
| Q43 | Other congenital malformations of intestine | 1 | 4 |
| G89 | Pain, not elsewhere classified | 1 | 1 |
| E03 | Other hypothyroidism | 1 | 1 |
| K50 | Crohn's disease [regional enteritis] | 1 | 1 |
| R10 | Abdominal and pelvic pain | 1 | 1 |
| T82 | Complications of cardiac and vascular prosthetic devices, implants and grafts | 1 | 1 |
| K43 | Ventral hernia | 1 | 2 |
| J98 | Other respiratory disorders | 1 | 1 |
| T81 | Complications of procedures, not elsewhere classified | 1 | 2 |
| N17 | Acute kidney failure | 1 | 1 |
| F50 | Eating disorders | 1 | 3 |
| R50 | Fever of other and unknown origin | 1 | 2 |
| E44 | Protein-calorie malnutrition of moderate and mild degree | 1 | 3 |
| F32 | Depressive episode | 1 | 2 |
| R00 | Abnormalities of heart beat | 1 | 2 |
| B37 | Candidiasis | 1 | 1 |
| I82 | Other venous embolism and thrombosis | 1 | 2 |
| E66 | Overweight and obesity | 1 | 1 |
| Q79 | Congenital malformations of musculoskeletal system, not elsewhere classified | 1 | 4 |

Abbreviations: ER, emergency room; ICD-10, International Statistical Classification of Diseases and Related Health Problems 10th Revision; SBS, short bowel syndrome.
